# Vaccine effectiveness for prevention of covid-19 related hospital admission during pregnancy in England during the alpha and delta variant dominant periods of the SARS-CoV-2 pandemic: population based cohort study

**DOI:** 10.1136/bmjmed-2022-000403

**Published:** 2023-07-10

**Authors:** Matthew L Bosworth, Ryan Schofield, Daniel Ayoubkhani, Loes Charlton, Vahé Nafilyan, Kamlesh Khunti, Francesco Zaccardi, Clare Gillies, Ashley Akbari, Marian Knight, Rachael Wood, Pia Hardelid, Luisa Zuccolo, Camille Harrison

**Affiliations:** 1 Office for National Statistics, Newport, UK; 2 Real World Evidence Unit, Diabetes Research Centre, University of Leicester, Leicester, UK; 3 London School of Hygiene and Tropical Medicine, London, UK; 4 Population Data Science, Swansea University Medical School, Swansea University, Swansea, UK; 5 National Perinatal Epidemiology Unit, Nuffield Department of Population Health, University of Oxford, Oxford, UK; 6 Public Health Scotland, Edinburgh, UK; 7 The University of Edinburgh Usher Institute of Population Health Sciences and Informatics, Edinburgh, UK; 8 NIHR Great Ormond Street Hospital Biomedical Research Centre, UCL Great Ormond Street Institute of Child Health, London, UK; 9 Health Data Science Centre, Fondazione Human Technopole, Milan, Italy; 10 MRC Integrative Epidemiology Unit, University of Bristol, Bristol, UK

**Keywords:** COVID-19, Public health, Epidemiology

## Abstract

**Objective:**

To estimate vaccine effectiveness for preventing covid-19 related hospital admission in individuals first infected with the SARS-CoV-2 virus during pregnancy compared with those of reproductive age who were not pregnant when first infected with the virus.

**Design:**

Population based cohort study.

**Setting:**

Office for National Statistics Public Health Data Asset linked dataset, providing national linked census and administrative data in England, 8 December 2020 to 31 August 2021.

**Participants:**

815 477 females aged 18-45 years (mean age 30.4 years) who had documented evidence of a first SARS-CoV-2 infection in the NHS Test and Trace or Hospital Episode Statistics data.

**Main outcome measures:**

Hospital admission where covid-19 was recorded as the primary diagnosis. Cox proportional hazards models, adjusted for calendar time of infection, sociodemographic factors, and pre-existing health conditions related to uptake of the covid-19 vaccine and risk of severe covid-19 outcomes, were used to estimate vaccine effectiveness as the complement of the hazard ratio for hospital admission for covid-19.

**Results:**

Compared with pregnant individuals who were not vaccinated, the adjusted rate of hospital admission for covid-19 was 77% (95% confidence interval 70% to 82%) lower for pregnant individuals who had received one dose and 83% (76% to 89%) lower for those who had received two doses of vaccine. These estimates were similar to those found in the non-pregnant group: 79% (77% to 81%) for one dose and 83% (82% to 85%) for two doses of vaccine. Among those who were vaccinated >90 days before infection, having two doses of vaccine was associated with a greater reduction in risk than one dose.

**Conclusions:**

Covid-19 vaccination was associated with reduced rates of hospital admission in pregnant individuals infected with the SARS-CoV-2 virus, and the reduction in risk was similar to that in non-pregnant individuals. Waning of vaccine effectiveness occurred more quickly after one than after two doses of vaccine.

**WHAT IS ALREADY KNOWN ON THIS TOPIC**

- Pregnancy is a risk factor for severe illness and death after infection with the SARS-CoV-2 virus
- Evidence suggests that covid-19 vaccines are effective for preventing severe outcomes in pregnant individuals
- Research directly comparing vaccine effectiveness between pregnant and non-pregnant individuals of reproductive age at the population level are lacking

**WHAT THIS STUDY ADDS**

- This study provides real world evidence that covid-19 vaccination reduced the risk of hospital admission by a similar amount in both individuals infected with the SARS-CoV-2 virus during pregnancy and in those who were not pregnant when infected, during the alpha and delta variant dominant periods of the covid-19 pandemic in England

**HOW THIS STUDY MIGHT AFFECT RESEARCH, PRACTICE, OR POLICY**

- This study adds to the body of evidence demonstrating effectiveness of covid-19 vaccination for reducing the risk of severe illness among those infected with SARS-CoV-2 during pregnancy
- Further research is needed to understand waning effectiveness of covid-19 booster vaccines in pregnancy

## Introduction

Physiological changes that take place during pregnancy (eg, insulin resistance, low blood pressure, and changes to respiration) increase the risk of experiencing severe outcomes of covid-19.^1^ Although the absolute risk of being admitted to hospital or death related to covid-19 during pregnancy is low,^2–4^ SARS-CoV-2 infection in pregnancy is associated with maternal and perinatal morbidity and mortality.^5–7^ A meta-analysis of 21 studies reported that pregnant and postpartum individuals with covid-19 were at increased risk of admission to the intensive care unit and all cause mortality than those not infected with the SARS-CoV-2 virus.^8^

Covid-19 vaccines have been shown to be highly effective at reducing the risk of hospital admission and death related to covid-19 in clinical trials and real world observational studies.^9–11^ Although pregnant individuals were not included in the original trials, a meta-analysis of three observational studies found that two doses of a mRNA vaccine was 89.5% effective in preventing SARS-CoV-2 infection during pregnancy.^12^ Other studies have shown that vaccination reduces the risk of severe illness in pregnant individuals infected with the SARS-CoV-2 virus.^13 14^ Consistent with these observations, most pregnant individuals admitted to hospital or intensive care units for covid-19 in the UK and Europe were not vaccinated.^15–17^ Despite accumulating evidence for the efficacy and safety of covid-19 vaccines in pregnancy, vaccine hesitancy remains high.^18^

An observational cohort study reported that two doses of the Pfizer-BioNTech (BNT162b2) mRNA vaccine was 89% effective in preventing covid-19 related hospital admissions in pregnant individuals during the periods when the wild-type and alpha variants of the SARS-CoV-2 virus were predominant in Israel, which was similar to the estimated efficacy in the general population.^19^ In another study from Israel, two or three doses of the mRNA vaccine were 96% and 99% effective, respectively, in preventing severe disease in pregnancy during the delta variant dominant period, decreasing to 83% and 94% during the omicron period.^20^ Large scale studies directly comparing vaccine effectiveness between pregnant and non-pregnant individuals of reproductive age at the population level after adjusting for sociodemographic characteristics linked with severe illness and vaccine uptake are, however, lacking. In this study, we used population level linked administrative data for England to estimate vaccine effectiveness in preventing hospital admission for covid-19 among those infected with the SARS-CoV-2 virus during pregnancy compared with a group who were not pregnant when infected with the virus.

## Methods

### Study data

We conducted a population based cohort study with data from the Office for National Statistics Public Health Data Asset. The Office for National Statistics Public Health Data Asset is a linked dataset combining data from the 2011 census of England, mortality records, the General Practice Extraction Service Data for Pandemic Planning and Research (GDPPR), Hospital Episode Statistics, vaccination data from the National Immunisation Management System, and data from the NHS Test and Trace pillar 1 (swab testing for the virus in UK Health Security Agency laboratories and NHS hospitals for those with a clinical need, and health and care workers) and pillar 2 (swab testing for the virus in the wider population, through commercial partnerships, either processed in a laboratory or more rapidly by lateral flow device tests).^21^ All data sources were accessed on 28 June 2022, except for GDPPR, which was accessed on 3 February 2022.

To obtain NHS numbers, data from the 2011 census were linked to the 2011-13 NHS patient registers with deterministic and probabilistic matching, with an overall linkage rate of 94.6% (detailed description of the linkage methodology and quality evaluation have been previously reported^22^). Further linkage to death registrations data, and GDPPR, Hospital Episode Statistics, and National Immunisation Management System data was performed deterministically with a unique identifier (NHS number).

We linked the Public Health Data Asset to data from NHS birth notifications for 2020, 2021, and January-March 2022, based on mothers’ NHS numbers. The birth notification is a document completed by the doctor or midwife present at the birth and is used to notify registration offices of the birth and issue NHS numbers to babies. Birth notifications data only include pregnancies resulting in a live birth or stillbirth after at least 24 weeks of gestation. Small differences exist in the number of births recorded between birth notifications and registrations data, but the two data sources are similar.^23^

We used data from the 2021 census of England for more up-to-date sociodemographic characteristics for participants in the study. The 2021 census was deterministically linked to the NHS Personal Demographics Service to retrieve NHS numbers, with a linkage rate of 94.6%. After clerical review of the links made, precision (proportion of true links) was estimated as 99.4% (95% confidence interval 96.5% to 100.0%); 1.6% of these links involved multiple 2021 census records linked to the same NHS number, which were excluded after deduplication. The 2021 census was then linked to the Public Health Data Asset with NHS numbers.

### Study population and design

The study cohort comprised females who had a first recorded SARS-CoV-2 infection between 8 December 2020 (the start of the vaccination campaign in the UK) and 31 August 2021 (with no evidence of a previous infection) and were listed in the 2011 census and living in a private household; aged 18-45 years at the start of the study period; linked to the 2011-13 NHS patient registers; linked to at least one GDPPR record; and resident in England according to the most recent postcodes held in GDPPR. Data for sex were taken from self-reported responses to the following question at the 2011 census: "What is your sex?"; response options were "male" or "female."

The index date for the start of follow-up was the earliest evidence of SARS-CoV-2 infection within the study period. Evidence of SARS-CoV-2 infection was determined by a positive swab test result for the SARS-CoV-2 virus with a polymerase chain reaction (PCR) test or a lateral flow device recorded in the NHS Test and Trace data, or a hospital admission or outpatient appointment with an ICD-10 (international classification of diseases, 10th revision) code of U07.1 (covid-19, virus identified) or U07.2 (covid-19, virus not identified) as the primary or secondary diagnosis.

### Method for identifying pregnancies

**Figure 1:**
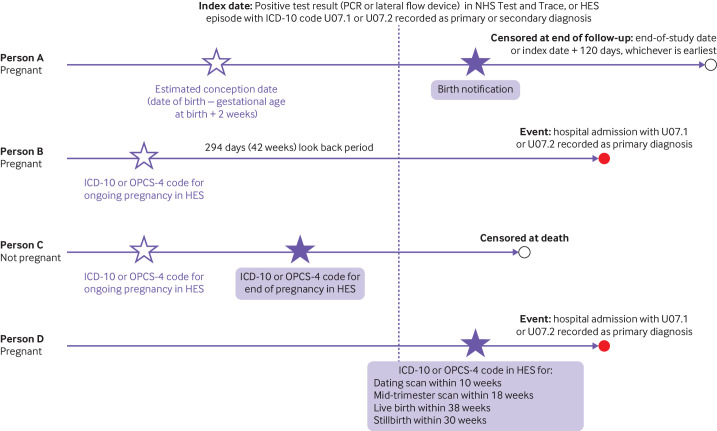
Method for identifying individuals who were pregnant when infected with the SARS-CoV-2 virus. PCR=polymerase chain reaction; HES=Hospital Episode Statistics; ICD-10=international classification of diseases, 10th revision; OPCS-4=Office of Population Censuses and Surveys Classification of Interventions and Procedures, version 4

Two data sources were used to identify pregnancy status at the time of SARS-CoV-2 infection. NHS birth notifications data identified those who were pregnant when infected with the SARS-CoV-2 virus and had a live birth or stillbirth. Hospital Episode Statistics data identified individuals who were pregnant when they were infected with the SARS-CoV-2 virus but for whom a birth notification was not recorded (eg, pregnancies that ended before 24 weeks which are not recorded in the birth notifications data, or pregnancies that were ongoing at the end of the study period). Figure 1 provides an overview of the methodology, and online supplemental materials has a more detailed description.

10.1136/bmjmed-2022-000403.supp1 Supplementary data

### Vaccination status

Vaccination status was derived from data from the National Immunisation Management System and was defined as the number of doses received at least 14 days before the index date. Participants were classified as single or double vaccinated if they had received one or two doses, respectively, of a covid-19 vaccine at least 14 days before infection.

### Covariates

We adjusted for sociodemographic factors known from previous studies to be associated with the risk of severe covid-19 outcomes, and vaccine uptake (online supplemental table S1 describes the data sources for each of the covariates).^24–28^ For 91.6% of participants, the covariates included from the 2021 census were age, ethnic group, English language proficiency, country of birth, keyworker status, highest educational qualification, disability status, and health status. For the remaining 8.4% of participants that could not be linked to the 2021 census, these variables were based on the 2011 census data. Missing census responses were imputed with nearest neighbour donor imputation.^29^

Geographical covariates were derived from postcodes in the GDPPR data. Region and rural-urban classifications were from the National Statistics Postcode Lookup.^30^ The index of multiple deprivation was from the English indices of deprivation 2019.^31^ We used GDPPR records (8 December 2000 to 8 December 2020) to derive pre-existing health conditions (online supplemental table S1). Health conditions were selected based on the QCOVID risk prediction model,^32^ which has been previously shown to predict the risk of severe covid-19 outcomes in three independent data sources.^33–35^ QCOVID was used in the UK to prioritise clinically vulnerable people for vaccination. We grouped the number of health conditions (0, 1, or ≥2) because of the small sample sizes for some health conditions.

### Outcome

The outcome was hospital admission with an ICD-10 code of U07.1 or U07.2 recorded as the primary diagnosis and occurring within 120 days of the index date; this time frame was used to avoid inclusion of outcomes related to a subsequent infection.^36^

### Statistical analysis

We calculated age standardised rates of hospital admission for covid-19 (per 100 000 infections) by vaccination status and pregnancy status, standardised to the 2013 European Standard Population.^37^ We used Cox proportional hazards models to assess how the rate of hospital admission for covid-19 varied by vaccination status (reference=unvaccinated group). Models were stratified by pregnancy status at the time of the first SARS-CoV-2 infection. Follow-up time was calculated from first infection (index date) until covid-19 related hospital admission, death, or 120 days of follow-up, whichever occurred first. The proportional hazards assumption was assessed by inspecting plots of the Schoenfeld residuals. We calculated hazard ratios adjusted for all sociodemographic covariates, pre-existing health conditions, and calendar time of infection to account for differences in the SARS-CoV-2 variant, changes in treatment strategies, and changes in hospital capacity over the study period (online supplemental table S1 describes how variables were modelled). Vaccine effectiveness was calculated as the complement of the hazard ratio.

To assess differential waning of vaccine effectiveness between the single and double vaccinated individuals, we grouped the analysis by time since last vaccine dose (14-90 days *v* >90 days^38 39^). We conducted a sensitivity analysis excluding 1671 particpants (5.0% of the total pregnant population) who were infected with SARS-CoV-2 before 11 June 2021 (42 weeks before 31 March 2022, the most recent birth notifications data available) who were identified as pregnant in the Hospital Episode Statistics data but not in the birth notifications data. These individuals might have been pregnant previously but were no longer pregnant when they were infected because the pregnancy ended early and was not recorded in the Hospital Episode Statistics records or birth notifications data. All statistical analyses were conducted with R version 3.5. Cox proportional hazards models were implemented with the survival package (version 2.41-3).^40^

### Patient and public involvement

We did not directly involve patients and the public in the design and conception of the study, primarily because of the pace at which this study was conducted to inform the UK government’s response to the covid-19 pandemic. The use of deidentified data precludes direct dissemination to participants.

## Results

### Characteristics of the study population

The study population included 815 477 females aged 18-45 years (mean 30.4, standard deviation 8.1 years); 33 549 (4.1%) were identified as pregnant when they were infected with the SARS-CoV-2 virus (table 1).

**Table 1:** Personal characteristics of study population

Variable	Non-pregnant group (n=781 928)			Pregnant group (n=33 549)		
	Not vaccinated (n=536 110)	Single vaccinated (n=128 317)	Double vaccinated (n=117 501)	Not vaccinated (n=29 244)	Single vaccinated (n=2733)	Double vaccinated (n=1572)
Mean (SD) age (years) on 8 December 2020	30.2 (8.1)	29.2 (8.1)	33.2 (8.3)	29.2 (5.5)	30.0 (5.5)	30.6 (5.7)
Mean (SD) calendar time of infection (days since 8 December 2020)	85.4 (84.9)	210.2 (51.1)	233.1 (23.0)	111.0 (93.8)	222.4 (37.8)	235.2 (21.3)
English region:						
North East	27 235 (5.1)	9140 (7.1)	8740 (7.4)	1725 (5.9)	210 (7.7)	130 (8.3)
North West	77 800 (14.5)	19 440 (15.1)	19 260 (16.4)	4540 (15 5)	415 (15.2)	275 (17.5)
Yorkshire and the Humber	49 075 (9.2)	16 070 (12.5)	15 515 (13.2)	2935 (10.0)	335 (12.3)	200 (12.7)
East Midlands	42 635 (8.0)	12 120 (9.4)	11 855 (10.1)	2375 (8.1)	270 (9.9)	165 (10.5)
West Midlands	59 250 (11.1)	12 930 (10.1)	12 415 (10.6)	3690 (12.6)	360 (13.2)	180 (11.5)
East of England	59 460 (11.1)	11 490 (9.0)	10 165 (8.7)	3265 (11.2)	240 (8.8)	120 (7.6)
London	103 850 (19.4)	14 130 (11.0)	11 680 (9.9)	4660 (15.9)	235 (8.6)	170 (10.8)
South East	81 715 (15.2)	18 585 (14.5)	15 285 (13.0)	4330 (14.8)	390 (14.3)	170 (10.8)
South West	35 095 (6.5)	14 410 (11.2)	12 580 (10.7)	1730 (5.9)	280 (10.2)	165 (10.5)
Rural and urban classification:						
Major conurbations	231 200 (43.1)	43 440 (33.9)	39 290 (33.4)	12 255 (41.9)	905 (33.1)	525 (33.4)
Minor conurbations	19 610 (3.7)	6335 (4.9)	6040 (5.1)	1130 (3.9)	130 (4.8)	75 (4.8)
Cities and towns	223 180 (41.6)	58 680 (45.7)	54 170 (46.1)	12 235 (41.8)	1225 (44.8)	735 (46.8)
Towns and fringes	34 650 (6.5)	10 860 (8.5)	10 350 (8.8)	2100 (7.2)	270 (9.9)	135 (8.6)
Villages, hamlets, and other isolated dwellings	27 465 (5.1)	9005 (7.0)	7650 (6.5)	1530 (5.2)	205 (7.5)	105 (6.7)
Index of multiple deprivation (10 groups):						
1 (most deprived)	67 025 (12.5)	12 960 (10.1)	12 945 (11.0)	4230 (14.5)	320 (11.7)	200 (12.7)
2	68 545 (12.8)	12 920 (10.1)	12 080 (10.3)	3815 (13.0)	270 (9.9)	180 (11.5)
3	64 680 (12.1)	13 275 (10.3)	11 865 (10.1)	3495 (12.0)	285 (10.4)	155 (9.9)
4	58 080 (10.8)	12 885 (10.0)	12 095 (10.3)	3115 (10.7)	250 (9.1)	160 (10.2)
5	53 825 (10.0)	13 100 (10.2)	11 375 (9.7)	2855 (9.8)	280 (10.2)	145 (9.2)
6	50 520 (9.4)	12 870 (10.0)	11 645 (9.9)	2690 (9.2)	280 (10.2)	165 (10.5)
7	46 735 (8.7)	12 700 (9.9)	11 595 (9.9)	2505 (8.6)	300 (11.0)	150 (9.5)
8	45 780 (8.5)	12 610 (9.8)	11 765 (10.0)	2405 (8.2)	250 (9.1)	160 (10.2)
9	43 205 (8.1)	12 490 (9.7)	11 415 (9.7)	2265 (7.7)	270 (9.9)	135 (8.6)
10 (least deprived)	37 720 (7.0)	12 510 (9.7)	10 725 (9.1)	1870 (6.4)	230 (8.4)	125 (8.0)
Ethnic group:						
Asian	64 875 (12.1)	7915 (6.2)	7900 (6.7)	3410 (11.7)	180 (6.6)	110 (7.0)
Black	28 005 (5.2)	2110 (1.6)	1700 (1.4)	1215 (4.2)	30 (1.1)	15 (1.0)
Mixed	20 785 (3.9)	3575 (2.8)	2635 (2.2)	975 (3.3)	65 (2.4)	50 (3.2)
White	411 420 (76.7)	113 575 (88.5)	103 990 (88.5)	23 140 (79.1)	2435 (89.1)	1390 (88.4)
Other	11 025 (2.1)	1140 (0.9)	1280 (1.1)	505 (1.7)	30 (1.1)	10 (0.6)
English is main language:						
Yes	498 870 (93.1)	124 240 (96.8)	113 680 (96.7)	27 785 (95.0)	2665 (97.5)	1540 (98.0)
No	37 240 (6.9)	4075 (3.2)	3825 (3.3)	1460 (5.0)	65 (2.4)	30 (1.9)
Born in the UK:						
Yes	459 485 (85.7)	118 085 (92.0)	107 830 (91.8)	25 980 (88.8)	2570 (94.0)	1480 (94.1)
No	76 625 (14.3)	10 230 (8.0)	9670 (8.2)	3265 (11.2)	160 (5.9)	90 (5.7)
Keyworker*:						
Yes	41 995 (7.8)	9075 (7.1)	11 875 (10.1)	2545 (8.7)	265 (9.7)	195 (12.4)
No	494 115 (92.2)	119 240 (92.9)	105 625 (89.9)	26 695 (91.3)	2465 (90.2)	1380 (87.8)
Highest educational qualification:						
Degree or above	194 580 (36.3)	48 375 (37.7)	50 815 (43.2)	11 240 (38.4)	1270 (46.5)	795 (50.6)
≥2 A levels or equivalent	139 610 (26.0)	35 850 (27.9)	30 340 (25.8)	7125 (24.4)	650 (23.8)	355 (22.6)
≥5 GCSE passes or equivalent	77 870 (14.5)	18 600 (14.5)	16 010 (13.6)	4300 (14.7)	355 (13.0)	180 (11.5)
1-4 GCSE passes or equivalent	39 095 (7.3)	7710 (6.0)	7580 (6.5)	2295 (7.8)	175 (6.4)	95 (6.0)
Apprenticeship or other qualification	22 810 (4.3)	4640 (3.6)	4000 (3.4)	1415 (4.8)	95 (3.5)	60 (3.8)
No qualifications	33 450 (6.2)	5575 (4.3)	5355 (4.6)	1925 (6.6)	125 (4.6)	65(4.1)
Not classified	28 695 (5.4)	7565 (5.9)	3400 (2.9)	940 (3.2)	65 (2.4)	25 (1.6)
Disability:						
None	471 065 (87.9)	114 120 (88.9)	99 045 (84.3)	26 615 (91.0)	2455 (89.8)	1360 (86.5)
Some limitation	47 580 (8.9)	11 175 (8.7)	13 700 (11.7)	1995 (6.8)	215 (7.9)	165 (10.5)
Severe limitation	17 465 (3.3)	3025 (2.4)	4755 (4.0)	630 (2.2)	65 (2.4)	50 (3.2)
Health status:						
Very good or good	483 445 (90.2)	118 760 (92.6)	102 570 (87.3)	27 210 (93.0)	2535 (92.8)	1395 (88.7)
Fair	41 650 (7.8)	7710 (6.0)	11 435 (9.7)	1690 (5.8)	170 (6.2)	140 (8.9)
Poor or very poor	11 020 (2.1)	1845 (1.4)	3500 (3.0)	345 (1.2)	25 (0.9)	35 (2.2)
No of pre-existing health conditions:						
0	442 720 (82.6)	109 085 (85.0)	88 250 (75.1)	24 315 (83.1)	2230 (81.6)	1180 (75.1)
1	87 285 (16.3)	18 450 (14.4)	26 690 (22.7)	4705 (16.1)	485 (17.7)	365 (23.2)
>2	6105 (1.1)	780 (0.6)	2560 (2.2)	225 (0.8)	20 (0.7)	25 (1.6)

Data are number (%) unless indicated otherwise. GCSE=General Certificate of Secondary Education. Online supplemental table S1 shows the data source for each variable. Counts are rounded to the nearest five to ensure that individuals cannot be identified in the data and percentages have been calculated with the rounded counts.

*Based on Standard Occupational Classification and the 2007 Standard Industrial Classification of Economic Activities. Keyworkers include the occupations education and childcare, national and local government, public safety and national security, food and necessity goods, utilities, and communication, transport, health and social care, and key public services.

Among those identified as pregnant at the time of SARS-CoV-2 infection, 87.2% had not received a vaccine, 8.1% were single vaccinated, and 4.7% were double vaccinated. Among non-pregnant individuals, 68.6% had not received a vaccine, 16.4% were single vaccinated, and 15.0% were double vaccinated.

### Age standardised rates of covid-19 related hospital admission

Overall, 9889 hospital admissions for covid-19 occurred in the study period: 1895 (19.2%) were among pregnant individuals (table 2), and 1807 (95.4%) of these had not received a vaccine; and 7994 (80.8%) occurred in non-pregnant individuals, of whom 7028 (87.9%) had not received a vaccine.

**Table 2:** Number and age standardised rates (per 100 000 infections) of covid-19 related hospital admissions by pregnancy and vaccination status when first infected with SARS-CoV-2 virus

Vaccination status	No of covid-19 related hospital admissions	Age standardised rate per 100 000 infections (95% CI)
Non-pregnant group (n=781 928):		
Not vaccinated	7028	1488 (1453 to 1523)
Single vaccinated	435	422 (380 to 463)
Double vaccinated	531	435 (398 to 473)
Pregnant group (n=33 549):		
Not vaccinated	1807	6737 (6253 to 7220)
Single vaccinated	60	2182 (1433 to 3090)
Double vaccinated	28	1590 (901 to 2504)

CI=confidence interval.

For both pregnant and non-pregnant individuals, age standardised rates of covid-19 related hospital admission (per 100 000 infections) were higher among those who had not received a vaccine compared with those who were single or double vaccinated (table 2). Among pregnant individuals, the age standardised rate of hospital admission for covid-19 was 6737 (95% confidence interval 6253 to 7220) per 100 000 infections for those who were not vaccinated, 2182 (1433 to 3090) for those who were single vaccinated, and 1590 (901 to 2504) for those who were double vaccinated. The corresponding rates for non-pregnant individuals were 1488 (1453 to 1523), 422 (380 to 463), and 435 (398 to 473), respectively.

### Vaccine effectiveness for preventing covid-19 related hospital admission

**Figure 2:**
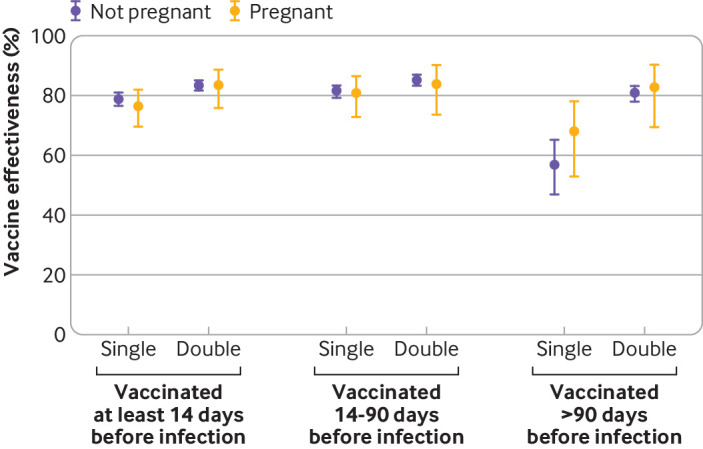
Vaccine effectiveness for preventing covid-19 related hospital admission, grouped by pregnancy status and time since vaccination. Estimates were calculated as the complement of the hazard ratio from Cox proportional hazards models adjusted for age, calendar time of infection, region, index of multiple deprivation group (10 groups), rural-urban classification, ethnic group, English language proficiency, country of birth, keyworker status, highest educational qualification, disability status, self-reported health status, and number of pre-existing health conditions. Analyses grouped by time since vaccination are subgroup analyses of the whole sample

Compared with the non-vaccinated pregnant group, vaccine effectiveness for preventing covid-19 related hospital admission was 77% (95% confidence interval 70% to 82%) for the single vaccinated pregnant group and 83% (76% to 89%) for the double vaccinated pregnant group (figure 2). Corresponding estimates for non-pregnant individuals were 79% (77% to 81%) and 83% (82% to 85%), respectively.

Analysis of Schoenfeld residuals plots indicated potential non-proportional hazards for vaccination after about four weeks of follow-up (online supplemental figure S1). We therefore conducted a sensitivity analysis restricted to 28 days of follow-up (covering 96.9% of hospital admissions included in the main analysis) and the results were not substantially different (online supplemental table S2). A further sensitivity analysis with logistic regression also showed almost identical results (online supplemental table S3). Results were also robust when 1671 participants who were potentially misclassified as pregnant were excluded from the analysis (online supplemental table S4).

In the pregnant and non-pregnant groups, vaccine effectiveness was similar in those single or double vaccinated 14-90 days before the first infection (figure 2). Among those vaccinated >90 days before the first infection, vaccine effectiveness was lower for the single vaccinated non-pregnant group (57%, 95% confidence interval 47% to 65%) than the double vaccinated non-pregnant group (81%, 78% to 83%) (P<0.001). A similar pattern was seen in the pregnant group (single vaccinated 68%, 53% to 78%; double vaccinated 83%, 69% to 90%) (P=0.07).

## Discussion

### Summary of main findings

Our study showed that vaccination against covid-19 was associated with a reduced risk of covid-19 related hospital admission during the periods when the alpha and delta variants of the SARS-CoV-2 virus were predominant in England, in individuals of reproductive age, regardless of their pregnancy status when infected with the virus. The effectiveness of the covid-19 vaccines against hospital admission waned more quickly after one than after two doses of vaccine in both pregnant and non-pregnant individuals.

### Comparison with other studies

Our findings are consistent with other studies showing that covid-19 vaccination is effective in reducing the risk of severe illness after infection with the SARS-CoV-2 virus in pregnancy.^13 14^ Dagan et al^19^ found that two doses of the Pfizer-BioNTech mRNA vaccine was 89% effective in preventing covid-19 related hospital admission in pregnant individuals during the wild-type and alpha variant dominant periods of the pandemic in Israel.^19^ Another study from Israel found that two doses of the Pfizer-BioNTech mRNA vaccine were 61% effective in preventing hospital admission with confirmed SARS-CoV-2 infection and 96% effective in preventing severe disease during pregnancy when the delta variant was the predominant strain of the virus.^20^ We found that two doses of vaccine was 83% effective in preventing hospital admissions when covid-19 was recorded as the primary condition being treated (or was responsible for the primary condition being treated) in participants who were pregnant when infected with the SARS-CoV-2 virus during the alpha and delta variant dominant periods combined. This result was after adjusting for a range of sociodemographic characteristics and pre-existing health conditions associated with the risk of severe illness and vaccine uptake.

We also found that having two doses of a covid-19 vaccine was associated with greater protection against hospital admission for covid-19 than one dose among those vaccinated >90 days before infection, suggesting faster waning of vaccine effectiveness after one dose of vaccine. The recommended interval between first and second doses of vaccine is 84 days. Participants who were single vaccinated >90 days before infection might have had to delay their second vaccination because they were infected with the SARS-CoV-2 virus (in the UK, a vaccine cannot be given within 28 days of a positive SARS-CoV-2 test result). Alternatively, participants might have decided to delay their second dose of the vaccine if they had an adverse reaction to the first dose or because of concerns about the safety of the vaccine if they discovered they were pregnant after their first dose. Because data from the National Immunisation Management System cover England only, some participants could have received their second dose of vaccine outside of England and been misclassified as single vaccinated when they were infected. This number is likely to be small, however; 94.9% of participants who were single vaccinated when they were infected had a second dose recorded in the National Immunisation Management System data at a later date.

Our study did not assess effectiveness against the omicron variant of the virus or the effectiveness of booster vaccines. Previous evidence suggests that three doses of vaccine are more effective than two, however, for preventing severe illness in pregnancy after infection during the omicron variant period.^20 41^ None of the pregnant individuals admitted to the intensive care unit for covid-19 in the UK during the omicron dominant variant period had received three doses of vaccine.^42^ Considering evidence in the general population that the effectiveness of three doses for preventing severe illness after infection with omicron wanes over time,^43 44^ future research should compare the waning of effectiveness after three doses of the vaccine in pregnant and non-pregnant groups.

### Strengths and limitations

The main strength of our study was that we used the nationwide linked data asset combining the 2011 and 2021 censuses of England, mortality records, hospital records, birth notifications data, vaccinations data, and SARS-CoV-2 testing data from national testing programmes. Based on hospital data and data from birth notifications, we identified individuals who were pregnant when they were infected with the SARS-CoV-2 virus. We adjusted for a range of sociodemographic characteristics and pre-existing health conditions associated with uptake of the vaccine and the risk of severe covid-19 outcomes. For most participants, sociodemographic variables were based on up-to-date data from the 2021 census.

A limitation of our study was that the study population might not fully represent the at risk population. The cohort did not include people living in England in 2011 who did not participate in the 2011 census (estimated to be 5% of households^45^); those who could not be linked to the 2011-13 NHS patient registers; those who immigrated since 2011; or those not registered with a general practitioner at the start of the covid-19 pandemic.

Misclassification of pregnancy status was possible because of limited data availability, especially in the first trimester and for those whose pregnancy ended before 24 weeks. Consequently, participants infected with the SARS-CoV-2 virus who had short pregnancies might have been misclassified as not pregnant. Conversely, participants who were no longer pregnant when they were infected with the SARS-CoV-2 virus might have been incorrectly classified as being pregnant. This misclassification could bias results in showing that the pregnant and non-pregnant groups were more similar than they actually were in terms of vaccine effectiveness. But this finding is likely to have introduced limited bias, however, because we found similar results in a sensitivity analysis excluding participants who were potentially misclassified as pregnant.

People with asymptomatic covid-19 might be less likely to use the NHS Test and Trace programme to test for SARS-CoV-2 infection. These people might also be less likely to report the result of the test. Data from the UK Coronavirus Infection Survey (where all study participants were tested for SARS-CoV-2 infection, irrespective of whether they have symptoms) suggested that about 40% of people who test positive for the SARS-CoV-2 virus do not develop symptoms within 35 days.^46^ In this study, evidence of SARS-CoV-2 infection was identified from national testing data, which means that asymptomatic infections were likely to be under-represented in the study population. Consequently, the rates of hospital admission for covid-19 reported here might be an overestimate of the true rates in the general population. Also, sociodemographic differences in covid-19 testing behaviours might also mean that some groups (eg, younger people, those from non-white ethnic groups, and people of lower socioeconomic status) are under-represented in our study.^47 48^

Although the precise real world sensitivity and specificity of reverse transcription PCR tests and rapid antigen tests for SARS-CoV-2 are not known, studies suggest that the false positive rate is generally low.^49 50^ More asymptomatic infections and false positive results might have been detected in the pregnant group, however, because of a combination of differences in SARS-CoV-2 testing behaviours during pregnancy and the requirement to undergo testing before antenatal appointments.

Hospital admissions for covid-19 were defined as inpatient admissions, where covid-19 was recorded as the primary diagnosis. These admissions will include some hospital admissions where the initial reason for admission was not related to covid-19, but the patient was subsequently diagnosed as having, and then treated for, covid-19 while in hospital. Hospital admissions for covid-19 will also include hospital acquired infections, which are probably more common among pregnant individuals who are more likely to have contact with hospitals than those in the general population.

### Implications

Pregnant individuals were identified as a vulnerable group and prioritised for covid-19 vaccination in December 2021 by the Joint Committee on Vaccination and Immunisation in the UK. The Royal College of Obstetricians and Gynaecologists strongly recommend that covid-19 vaccines are offered to all pregnant individuals.^51^ Several studies have shown a lower risk of stillbirths in those vaccinated and no evidence of adverse pregnancy outcomes after covid-19 vaccination.^12 52 53^ Vaccination coverage among individuals giving birth has been increasing over time, but uptake remains lower at the time of delivery in those from ethnic minority groups, with the lowest vaccination rates in black women and those living in deprived areas.^11^ Interventions to deal with these inequalities and engagement with individuals who are pregnant to ensure uptake of potential future booster vaccines are needed, because many who become pregnant might have received their last dose of a vaccine several months previously.

### Conclusions

During the periods when the alpha and delta variants of the SARS-CoV-2 were predominant in England, covid-19 vaccination was associated with a reduced risk of covid-19 related hospital admission in individuals infected with SARS-CoV-2 during pregnancy as well as among non-pregnant individuals. Increasing vaccine uptake during pregnancy might contribute to reduced levels of avoidable harms to pregnant individuals associated with covid-19. These data add to the evidence base about the protective effect of vaccination for those infected with SARS-CoV-2 during pregnancy by providing real world evidence in a population that was originally excluded from the vaccine trials.

## Data Availability

No data are available. In accordance with NHS Digital’s Information Governance requirements, the study data cannot be shared.
