# Supplementary material for: Vaccine effectiveness for prevention of covid-19 related hospital admission during pregnancy in England during the alpha and delta variant dominant periods of the SARS-CoV-2 pandemic: population based cohort study
Source: BMJ Med. 2023 Jul 10;2(1):e000403. doi: 10.1136/bmjmed-2022-000403 (PMC10410807; doi:10.1136/bmjmed-2022-000403)
Supplement: Supplementary data [file bmjmed-2022-000403supp001.pdf]

Supplementary Materials

Method for identifying pregnancy from birth notifications data

The estimated conception date was calculated by subtracting gestational age from the date of birth on the earliest birth notification occurring after the index date. Two weeks were added to account for the fact that gestational age is defined from the start of the last menstrual period, and conception is assumed to occur at the mid-way point (day 14) of a typically assumed average 28-day menstrual cycle. Women were classified as pregnant at the index date if there was evidence of SARS-CoV-2 infection between the estimated conception date and date of birth on the birth notification.

Gestational age was missing for 0.3% of the birth notifications used to estimate the conception date. For these records, gestational age was imputed as 40 weeks for live births and 32 weeks for stillbirths (the approximate average length of gestation for these outcomes, calculated from published births data [1]).

Method for identifying pregnancy from Hospital Episode Statistics (HES) data

(A) We searched HES data for hospital episodes with evidence of ongoing pregnancy and end of pregnancy in the 42 weeks before the index date (**Tables S5** to **S8** report the code lists used). Participants were classified as pregnant at the index date if they met all of the following criteria:

- They had at least one hospital episode with an ICD-10 or Classification of Interventions and Procedures version 4 (OPCS-4) code indicating pregnancy over the 42 weeks prior to and including the index date.
- The most recent ICD-10 or OPCS-4 code over the 42 weeks prior to the index date indicated ongoing pregnancy (not the end of pregnancy).
- There was no end of pregnancy code in the six weeks prior to the most recent ongoing pregnancy code (some of the ongoing pregnancy codes relate to conditions that can be diagnosed in the postpartum period, up to six weeks after delivery).

(B) We also searched HES data for hospital episodes with evidence of ongoing pregnancy (routine obstetric scans only) and birth events occurring after the index date. Participants were classified as pregnant at the index date if any of the following criteria were met:

- They had a hospital episode with an OPCS-4 code for a dating scan (R36.1; normally performed at week 12 of pregnancy) up to 70 days after the index date.
- They had a hospital episode with an OPCS-4 code for a mid-trimester scan (R36.3; normally performed at week 20 of pregnancy) up to 126 days after the index date.

- They had a hospital episode with an ICD-10 code for a live birth (Z37.0, Z37.2, Z37.5 or Z38) or mixed live and stillbirth (Z37.3 or Z37.6) up to 38 weeks after the index date.
- They had a hospital episode with an ICD-10 code for a stillbirth (Z37.1, Z37.4 or Z37.7) up to 30 weeks after the index date.

References

[1] Office for National Statistics, Birth characteristics dataset, 2020 edition, 2022. Available at: https://www.ons.gov.uk/peoplepopulationandcommunity/birthsdeathsandmarriages/livebirths/datasets/birthcharacteristicsinenglandandwales. [Accessed 1 2 2022].

Table S1. Coding and source of variables included in the analysis

**Variable**	**Coding**	**Source**
Vaccination status	Unvaccinated (reference group), single-vaccinated, double-vaccinated	NIMS
Pregnancy status	Pregnant, not pregnant	NHS birth notifications and HES
Age	Restricted cubic spline with an internal knot at the 50th percentile and boundary knots at the 10th and 90th percentiles	2021 Census and 2011 Census
Calendar time of infection	Restricted cubic spline with an internal knot at the 50th percentile and boundary knots at the 10th and 90th percentiles	NHS Test and Trace and HES
Region	North East, North West, Yorkshire and the Humber, East Midlands, West Midlands, East of England, London, South East, South West	Postcodes from GDPPR and NSPL
Rural Urban classification	Major conurbations, minor conurbations, cities and towns, towns and fringes, villages, hamlets and other isolated dwellings	Postcodes from GDPPR and NSPL
Index of Multiple Deprivation	Dummy variables representing deciles of deprivation	Postcodes from GDPPR and English Indices of Deprivation, 2019
Ethnic group	Asian, Black, Mixed, White, Other	2021 and 2011 Census
English language proficiency	Main language, speak English very well or well, do not speak English well or at all	2021 and 2011 Census
Country of birth	UK, non-UK	2021 and 2011 Census
Keyworker status	Binary flag	2021 and 2011 Census

Bosworth ML, *et al. BMJMED* 2023; 2:e000403. doi: 10.1136/bmjmed-2022-000403

Highest qualification held	Degree or above, two or more A-levels or equivalent, five or more GCSE passes or equivalent, one to four GCSE passes or equivalent, apprenticeship or other qualification, no qualifications	2021 and 2011 Census
Disability status	Not disabled, disabled and limited a little, disabled and limited a lot	2021 and 2011 Census
Health status	Very good, good, fair, bad, very bad	2021 and 2011 Census
Number of pre-existing health conditions	0, 1, ≥2 (asthma, atrial fibrillation, blood cancer, chronic kidney disease, congenital heart problem, chronic obstructive pulmonary disease, congestive cardiac failure, coronary heart disease, cystic fibrosis, epilepsy, learning disability, liver cirrhosis, peripheral vascular disease, pulmonary hypertension or fibrosis, respiratory cancer, rheumatoid arthritis or systemic lupus erythematosus, stroke, severe mental illness, sickle cell disease, type 1 or type 2 diabetes)	GDPPR (8th December 2000 to 8th December 2020)

GDPPR, General Practice Extraction Service Data for Pandemic Planning and Research; HES, Hospital Episode Statistics; NSPL, National Statistics Postcode Lookup; NIMS, National Immunisation Management Service

Bosworth ML, *et al. BMJMED* 2023; 2:e000403. doi: 10.1136/bmjmed-2022-000403

Table S2. Hazard ratios for COVID-19 hospital admission within 28 days of SARS-CoV-2 infection

Pregnancy status	Vaccination status	Hazard ratio[1] (95% CI)
Not pregnant	Single-vaccinated	0.21 (0.19 to 0.23)
	Double-vaccinated	0.16 (0.15 to 0.18)
Pregnant	Single-vaccinated	0.24 (0.18 to 0.31)
	Double-vaccinated	0.17 (0.12 to 0.25)

[1]Results obtained from Cox proportional hazards models adjusted for age, calendar time of infection, region, Index of Multiple deprivation decile group, Rural/Urban classification, ethnic group, English language proficiency, country of birth, keyworker status, highest qualification held, disability status, self-reported health status, and number of pre-existing conditions.

Bosworth ML, *et al. BMJMED* 2023; 2:e000403. doi: 10.1136/bmjmed-2022-000403

Table S3. Odds ratios for COVID-19 hospital admission within 120 days of SARS-CoV-2 infection

Pregnancy status	Vaccination status	Odds ratio[1] (95% CI)
Not pregnant	Single-vaccinated	0.21 (0.19 to 0.23)
	Double-vaccinated	0.16 (0.14 to 0.18)
Pregnant	Single-vaccinated	0.22 (0.17 to 0.29)
	Double-vaccinated	0.15 (0.10 to 0.23)

[1]Results obtained from binary logistic regression models adjusted for age, calendar time of infection, region, Index of Multiple deprivation decile group, Rural/Urban classification, ethnic group, English language proficiency, country of birth, keyworker status, highest qualification held, disability status, self-reported health status, and number of pre-existing conditions.

BMJ Publishing Group Limited (BMJ) disclaims all liability and responsibility arising from any reliance placed on this supplemental material which has been supplied by the author(s)

Table S4. Hazard ratios for COVID-19 hospital admission in pregnant women from sensitivity analysis excluding those who were potentially misclassified as pregnant

Vaccination status	Number of pregnant women	Hazard ratio[1] (95% CI)
Unvaccinated	27,629	Ref
Single-vaccinated	2,681	0.24 (0.18 to 0.31)
Double-vaccinated	1,568	0.17 (0.11 to 0.24)

[1]Results obtained from Cox proportional hazards models adjusted for age, calendar time of infection, region, Index of Multiple deprivation decile group, Rural/Urban classification, ethnic group, English language proficiency, country of birth, keyworker status, highest qualification held, disability status, self-reported health status, and number of pre-existing conditions.

Bosworth ML, *et al. BMJMED* 2023; 2:e000403. doi: 10.1136/bmjmed-2022-000403

Table S5. ICD-10 code list for ongoing pregnancy

Code	Description
O07%	Failed attempted abortion
O10%	Pre-existing hypertension complicating pregnancy, childbirth and the puerperium
O11%	Pre-eclampsia superimposed on chronic hypertension
O12%	Gestational oedema and proteinuria without hypertension
O13%	Gestational hypertension
O14%	Pre-eclampsia
O15%	Eclampsia
O16%	Unspecified maternal hypertension
O20%	Haemorrhage in early pregnancy
O21%	Excessive vomiting in pregnancy
O22%	Venous complications and haemorrhoids in pregnancy
O23%	Infections of genitourinary tract in pregnancy
O24%	Diabetes mellitus in pregnancy
O25%	Malnutrition in pregnancy
O26%	Maternal care for other conditions predominantly related to pregnancy
O28%	Abnormal findings on antenatal screening of mother
O29%	Complications of anaesthesia during pregnancy
O30%	Multiple gestation
O31%	Complications specific to multiple gestation
O32%	Maternal care for known or suspected malpresentation of fetus
O33%	Maternal care for known or suspected disproportion
O34%	Maternal care for known or suspected abnormality of pelvic organs
O35%	Maternal care for known or suspected fetal abnormality and damage
O36%	Maternal care for other known or suspected fetal problems
O40%	Polyhydramnios
O41%	Other disorders of amniotic fluid and membranes
O42%	Premature rupture of membranes
O43%	Placental disorders
O44%	Placenta praevia
O45%	Premature separation of placenta [abruptio placentae]
O46%	Antepartum haemorrhage, not elsewhere classified
O47%	False labour
O48%	Prolonged pregnancy
O88%	Obstetric embolism
O98%	Maternal infectious and parasitic diseases classifiable elsewhere but complicating pregnancy, childbirth and the puerperium
O99%	Other maternal diseases classifiable elsewhere but complicating pregnancy, childbirth and the puerperium
Z321	Pregnancy confirmed
Z33%	Pregnant state, incidental
Z34%	Supervision of normal pregnancy
Z35%	Supervision of high-risk pregnancy
Z36%	Antenatal screening

Bosworth ML, *et al. BMJMED* 2023; 2:e000403. doi: 10.1136/bmjmed-2022-000403

Table S6. OPCS-4 code list for ongoing pregnancy

Code	Description
R01%	Therapeutic endoscopic operations on fetus
R02%	Diagnostic endoscopic examination of fetus
R04%	Therapeutic percutaneous operations on fetus
R05%	Diagnostic percutaneous examination of fetus
R061	Selective feticide NEC
R063	Percutaneous selective feticide by vascular occlusion of umbilical cord
R07%	Therapeutic endoscopic operations for twin to twin transfusion syndrome
R08%	Therapeutic percutaneous operations for twin to twin transfusion syndrome
R09%	Open operations on fetus
R10%	Other operations on amniotic cavity
R11%	Other therapeutic percutaneous operations on fetus
R12%	Operations on gravid uterus
R13%	Other operations on fetus
R36%	Routine obstetric scan
R37%	Non-routine obstetric scan for fetal observations
R38%	Other non-routine obstetric scan
R40%	Other maternal physiological assessments
R42%	Obstetric Doppler ultrasound
R43%	Ultrasound monitoring

Bosworth ML, *et al. BMJMED* 2023; 2:e000403. doi: 10.1136/bmjmed-2022-000403

Table S7. ICD-10 code list for the end of pregnancy outcomes

Code	Description
O00%	Ectopic pregnancy
O01%	Hydatidiform mole
O02%	Other abnormal products of conception
O03%	Spontaneous abortion
O04%	Medical abortion
O05%	Other abortion
O06%	Unspecified abortion
O08%	Complications following abortion and ectopic and molar pregnancy
O60%	Preterm labour and delivery
O61%	Failed induction of labour
O62%	Abnormalities of forces of labour
O63%	Long labour
O64%	Obstructed labour due to malposition and malpresentation of fetus
O65%	Obstructed labour due to maternal pelvic abnormality
O66%	Other obstructed labour
O67%	Labour and delivery complicated by intrapartum haemorrhage, not elsewhere classified
O68%	Labour and delivery complicated by fetal stress [distress]
O69%	Labour and delivery complicated by umbilical cord complications
O70%	Perineal laceration during delivery
O71%	Other obstetric trauma
O72%	Postpartum haemorrhage
O73%	Retained placenta and membranes, without haemorrhage
O74%	Complications of anaesthesia during labour and delivery
O75%	Other complications of labour and delivery, not elsewhere classified
O80%	Single spontaneous delivery
O81%	Single delivery by forceps and vacuum extractor
O82%	Single delivery by caesarean section
O83%	Other assisted single delivery
O84%	Multiple delivery
O85%	Puerperal sepsis
O86%	Other puerperal infections
O87%	Venous complications and haemorrhoids in the puerperium
O89%	Complications of anaesthesia during the puerperium
O90%	Complications of the puerperium, not elsewhere classified
Z37%	Outcome of delivery
Z38%	Liveborn infants according to place of birth and type of delivery

Bosworth ML, *et al. BMJMED* 2023; 2:e000403. doi: 10.1136/bmjmed-2022-000403

Table S8. OPCS-4 code list for the end of pregnancy outcomes

Code	Description
Q091	Open removal of products of conception from uterus
Q101	Dilation of cervix uteri and curettage of products of conception from uterus
Q102	Curettage of products of conception from uterus NEC
Q111	Vacuum aspiration of products of conception from uterus NEC
Q112	Dilation of cervix uteri and evacuation of products of conception from uterus NEC
Q113	Evacuation of products of conception from uterus NEC
Q114	Extraction of menses
Q115	Vacuum aspiration of products of conception from uterus using rigid cannula
Q116	Vacuum aspiration of products of conception from uterus using flexible cannula
Q311	Removal of products of conception from fallopian tube
Q58%	Delivery of terminated fetus
R062	Feticide NEC
R14%	Surgical induction of labour
R15%	Other induction of labour
R17%	Elective caesarean delivery
R18%	Other caesarean delivery
R19%	Breech extraction delivery
R20%	Other breech delivery
R21%	Forceps cephalic delivery
R22%	Vacuum delivery
R23%	Cephalic vaginal delivery with abnormal presentation of head at delivery without instrument
R24%	Normal delivery
R25%	Other methods of delivery
R27%	Other operations to facilitate delivery
R28%	Instrumental removal of products of conception from delivered uterus
R29%	Manual removal of products of conception from delivered uterus
R30%	Other operations on delivered uterus
R32%	Repair of obstetric laceration

Bosworth ML, *et al. BMJMED* 2023; 2:e000403. doi: 10.1136/bmjmed-2022-000403

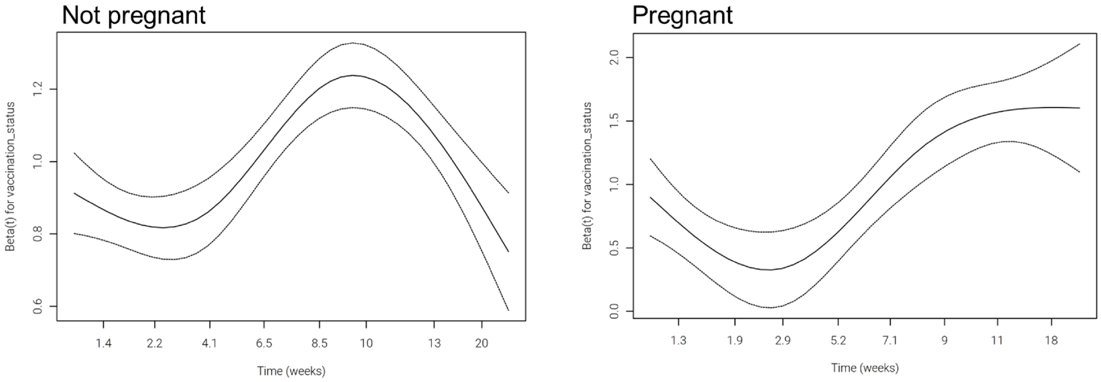

Figure S1. Schoenfeld residual plots for vaccination coefficient in non-pregnant (left) and pregnant (right) women.

Bosworth ML, *et al. BMJMED* 2023; 2:e000403. doi: 10.1136/bmjmed-2022-000403
